# Analyzing non‐cancer causes of death of colorectal carcinoma patients in the US population for the years 2000–2016

**DOI:** 10.1002/cam4.3673

**Published:** 2020-12-13

**Authors:** Lili Lu, Li Ma, Xianbin Zhang, Christina Susanne Mullins, Michael Linnebacher

**Affiliations:** ^1^ Department of General Surgery, Molecular Oncology and Immunotherapy Rostock University Medical Center Rostock Germany; ^2^ Department of Epidemiology Dalian Medical University Dalian China; ^3^ Department of General Surgery Shenzhen University General Hospital & Carson International Cancer Research Centre Shenzhen China

**Keywords:** age at diagnosis, colorectal cancer, cumulative incidence, follow‐up time, non‐cancer death, SMR

## Abstract

**Background:**

Colorectal cancer (CRC) treatment and patient survival improved greatly. Consequently an increased incidence of non‐cancer‐related deaths is observed. This study analyzed the causes of non‐cancer death for people suffering from CRC based on the year of diagnosis, follow‐up time, and patient's age.

**Methods:**

The data from patients diagnosed with CRC in the years 2000–2016 were taken from the Surveillance, Epidemiology, and End Results 18 database. Patients were categorized according to: death from CRC, non‐CRC cancer, and non‐cancer. Constituent ratios and standardized mortality ratios (SMRs) were calculated to describe the death causes distribution and relative death risks.

**Results:**

Between 2000 and 2016, a stable and rapid drop for the original diagnosis as death cause for CRC patients was observed (70.19% to 49.35%). This was coupled to an increase in non‐cancer‐associated death reasons (23.38% to 40.00%). The most common non‐cancer death cause was heart disease, especially for elderly patients. However, deaths from accidents and adverse effects were frequent in younger CRC patients. Patients died from septicemia more often within the first follow‐up year; however, a 6‐fold increase in death from Alzheimer's disease was found for after at least 180 months follow‐up time.

The SMRs of all 25 non‐cancer death causes initially decreased in all CRC subgroups, followed by an increase with follow‐up times. Gradually decreasing SMR values were observed with increasing age of CRC patients.

**Conclusions:**

These findings could help modify and sharpen preventive measures and clinical management and raise physician's awareness to potential non‐CRC death risk factors for CRC patients.

## Graphical abstract

The cumulative incidence of non‐cancer as cause for death among colorectal cancer (CRC) patients shows a stably increasing trend from 23.38% in 2000 to 40.00% in 2016 with non‐cancer death being mostly due to age‐associated diseases and attributable to the elevated average age of CRC patients. The higher relative risk of non‐cancer death for CRC patients is especially prominent within the first year of diagnosis, in the patient subgroup with very long survival time, and for younger CRC patients. This highlights the necessity for better individual management of CRC patients.

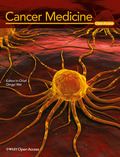

## 1 INTRODUCTION

In the United States, the recent cancer statistics showed that colorectal cancer (CRC) for both sexes combined still remains the second common cause of cancer‐related death. Projections estimate that approximately 147,950 individuals will be diagnosed with CRC and 53,200 will die from the disease in 2020.^1^

The incidence of CRC is still rapidly increasing in some developing countries, for example, in Eastern Europe as well as in Asia. However, in line with developments in other developed countries, such as Canada and several Northern European countries,^2^ a rapid decline in CRC incidence has been observed in the US with rates dropping by 2.5% per year from 2007 to 2016.^1^ Even more importantly, also mortality decreased by 2.1% annually during this period.^1^ When contemplating an even longer period, the 5‐year relative survival rate for CRC has increased from 50% in the mid‐1970's to 65% in 2016.^1^, ^3^ Various reasons contribute to this improvement in prognostic outcome including optimization of chemo‐ and radiation therapy as well as therapy adjustments. The most important factors hereby are age, tumor stage, comorbidities, and diagnosis year.^4^, ^5^, ^6^, ^7^, ^8^ As a result, patients with CRC survive substantially longer. A logical consequence of this favorable development is an increasing incidence of non‐cancer deaths among the cancer survivors. This has previously been analyzed for cancer patients diagnosed between 1973 and 2012.^9^The growing gap between overall and cancer‐specific survival is of high interest for clinical management of the increasing number of cancer survivors. As most common causes of non‐cancer death, chronic comorbidities and death from acute, iatrogenic, or therapeutic infections have been identified.^9^

When considering that heart disease is generally the first leading cause of death in the US,^10^ it comes to no surprise that this has also been described for elder US cancer patients.^9^ On the other hand, antitumoral treatments have been known to cause death from infections, accidents as well as suicide and self‐inflicted injury.^9^ Thus, it might be very important for individualized health care decision‐making to take into account that cancer patients are at a higher risk of dying for different non‐cancer reasons and that this risk depends on factors including patient's age, survival time, and treatments received.^11^

In the present study, we analyzed the most recent available population‐based data collected in the SEER database. The aims are first of all to describe the causes of non‐cancer death for patients diagnosed with CRC in the US during the period of 2000–2016. An additional aim is to evaluate the relative risk of non‐cancer death causes in comparison to the general US population based on follow‐up time as well as patient's age at diagnosis.

## 2 METHODS

### 2.1 Data source

We were allowed to utilize the Surveillance, Epidemiology, and End Results (SEER) stat client‐server system by signing the Research Data Agreement. The program collects incidence, mortality based on incidence, and survival data on cases. For the present study, the SEER 18 registries (2018 submission) were selected, which covers nearly 28% of the US population based on the 2010 census.

### 2.2 Study population

This study included cases from the SEER 18 registries with CRC diagnosed in the period of 2000–2016. We used topography codes (C18.0‐Cecum, C18.2‐Ascending colon, C18.3‐Hepatic flexure of colon, C18.4‐Transverse colon, C18.5‐Splenic flexure of colon, C18.6‐Descending colon, C18.7‐Sigmoid colon, C18.8‐Overlapping lesion of colon, C18.9‐Colon, NOS, C19.9‐Rectosigmoid junction, C20.9‐Rectum, NOS) combined with morphology code from the International Classification of Disease for Oncology codes, 3rd edition (8000–8152, 8154–8231, 8243–8245, 8247–8248, 8250–8934, 8940–9136, 9141–9582, 9700–9701) to identify CRC patients.^12^ Only patients with pathologically confirmed CRC 2 months later were included.^9^

### 2.3 Outcomes and variables

Death causes were obtained from *SEER cause of death recode* variable.^13^ SEER causes of death are recoded based on the International Statistical Classification of Diseases and Related Health Problems, 10^th^ Revision. In the current study, causes of death were categorized as CRC death (death due to colon and rectum cancer), non‐CRC cancer death (death due to cancer excluding colon and rectum cancer), and non‐cancer death (death due to any non‐cancer causes).

We evaluated these causes of non‐cancer death using the following variables: year of diagnosis (2000–2016), patients’ age at diagnosis (0–24, 25–29, 30–34, 35–39, 40–44, 45–49, 50–54, 55–59, 60–64, 65–69, 70–74, 75–79, 80–84, and ≥85 years old), and follow‐up time (2–11, 12–35, 36–59, 60–119, 120–179, and ≥180 months) after diagnosis.

### 2.4 Statistical analysis

To describe the distribution of non‐cancer death causes of CRC patients, we calculated the percentage of non‐cancer deaths within each year of diagnosis, age groups as well as follow‐up time. In order to evaluate the relative risk of non‐cancer death causes for CRC patients as compared to the general US population, we calculated the standardized mortality ratios (SMRs) which are defined as a ratio of the observed number of deaths in this CRC cohort to the expected number of deaths based on the age‐ and gender‐specific rates in the matched standard population (included in the SEER Stat 8.3.6 software). Observed value represents the number of CRC patients who died of a certain cause, and expected value represents the number of people who died of the same cause in the general population within the same time period analyzed and is calculated by multiplying the number of persons in each group of the study population by the age‐ and gender‐specific death rates of the general population.^14^, ^15^

For testing the significance of the observed SMRs, the simple continuity corrected Chi‐square statistic was used in order to test whether the observed number of deaths is significantly different from the expected number.^16^ A SMR greater than 1 indicates a higher relative risk of non‐cancer death causes in CRC patients versus the general US population. The observed and expected number of death, SMRs as well as 95% confidence interval (CI) of SMRs among CRC patients by each cause of death were obtained by taking advantage of SEER Stat 8.3.6 software.^17^ *p* < 0.05 was regarded as a statistical difference.

## 3 RESULTS

### 3.1 Death causes of CRC patients

**FIGURE 1:**
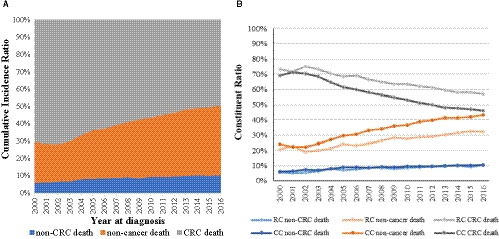
The trends of cumulative incidence ratio for death causes of (A) patients with CRC and (B) of patients with CC and RC during the period of 2000–2016

In total, 218,597 CRC patients died during the period of 2000–2016, consisting of 126,144 (57.71%) deaths from CRC cause, 19,574 (8.95%) deaths caused by any non‐CRC malignant cancer, and 72,219 (33.04%) deaths from non‐cancer causes (Table S1). Of those, 55,177 were colon cancer (CC) and 17,042 were rectal cancer (RC patients), 76.07% of the included CRC patients were 75 years or older at time of diagnosis, genders were evenly distributed, the majority were Caucasians, tumor stages were 52.19% localized, 38.33% regional, and 5.53% with distant metastases, and 91.79% patients received surgery (Table 1). Notably, the percentage of CRC patients dying not from their cancer disease—in other words, the cumulative incidence ratio of non‐cancer deaths among CRC cases—showed a stably increasing trend from 23.38% in 2000 to 40.00% in 2016 (Figure 1A and Table S1); this was more pronounced for CC patients with an increase from 24.19% to 43.19% compared to RC patients risk rising from 20.82% to 32.16% during the period of 2000–2016 (Figure 1B and Table S1).

**TABLE 1:** Clinicopathological characteristics of CRC patients died from non‐cancer causes during 2000–2016

Non‐cancer death						
Variables	CRC (n = 72,219)		CC (n = 55,177)		RC (n = 17,042)	
	n	%	n	%	n	%
Age at diagnosis (years old)						
≤49	719	0.996	463	0.839	256	1.502
50–74	16,563	22.934	11,441	20.735	5,122	30.055
≥75	54,937	76.070	43,273	78.426	11,664	68.443
Follow‐up time (months)						
2–11	12,285	17.011	9,149	16.581	3,136	18.402
12–35	15,067	20.863	11,293	20.467	3,774	22.145
36–59	12,104	16.760	9,411	17.056	2,693	15.802
60–119	22,357	30.957	17,419	31.569	4,938	28.975
120–179	9,661	13.377	7,368	13.353	2,293	13.455
≥180	745	1.032	537	0.973	208	1.221
Year at diagnosis						
2000–2005	12,120	16.782	9,124	16.536	2,996	17.580
2006–2011	28,004	38.776	21,404	38.792	6,600	38.728
2012–2016	32,095	44.441	24,649	44.673	7,446	43.692
Gender						
Male	36,346	50.327	26,490	48.009	9,856	57.834
Female	35,873	49.673	28,687	51.991	7,186	42.166
Race						
White	61,098	84.601	46,522	84.314	14,576	85.530
Black	7,276	10.075	5,824	10.555	1,452	8.520
A and PI	3,598	4.982	2,648	4.799	950	5.574
IA and AN	247	0.342	183	0.332	64	0.376
Tumor stage						
Localized	37,689	52.187	28,419	51.505	9,270	54.395
Regional	27,679	38.326	21,941	39.765	5,738	33.670
Distant	3,994	5.530	2,901	5.258	1,093	6.414
Unknown	2,857	3.956	1,916	3.472	941	5.522
Surgery						
Yes	66,292	91.793	51,929	94.113	14,363	84.280
No/unknown	5,927	8.207	3,248	5.887	2,679	15.720

### 3.2 Non‐cancer death causes of CRC patients

**FIGURE 2:**
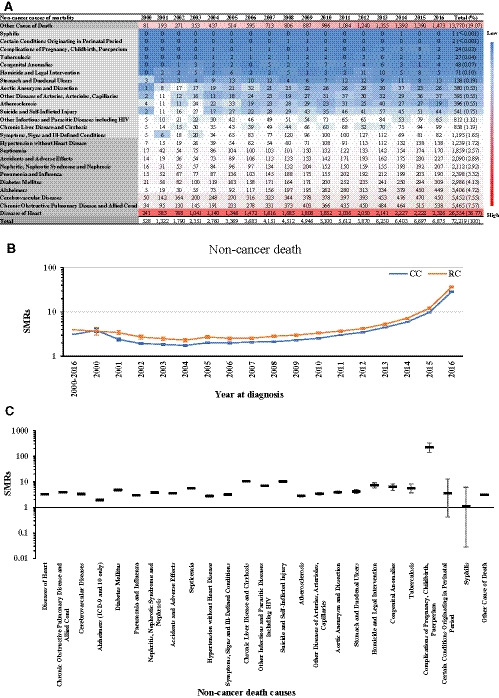
(A) The death number per cause of non‐cancer death for CRC patients during the period of 2000–2016. (B) The SMR values of non‐cancer death for patients with CC and RC for the years 2000–2016. (C) The SMR values per cause of non‐cancer death for CRC patients for the years 2000–2016. Horizontal lines are identified as SMR (middle) and 95% CI (upper: upper CI; lower: lower CI)

Based on the number of deaths per cause of non‐cancer death during the period of 2000–2016, we next sorted by the causes of non‐cancer death. The, by far, most common non‐cancer death cause was heart disease accounting for 36.77% of all non‐cancer deaths for CRC patients, followed by chronic obstructive pulmonary disease and allied conditions with 7.57% and cerebrovascular disease with 7.55%. The more detailed data can be depicted from Figure 2A and Table S2.

During the period of 2000–2016, the SMRs of non‐cancer death among CC and RC cases were 3.14 (95% CI, 3.12–3.17; *p* < 0.05) and 3.97 (95% CI, 3.91–4.03; *p* < 0.05), respectively, and showed a stably increasing trend from 2006 to 2016. Notably, the SMRs had reached 28.48 (95% CI, 27.71–29.26; *p* < 0.05) for CC and 36.40 (95% CI, 34.64–38.24; *p* < 0.05) for RC by 2016 (Figure 2B and Table S2).

This analysis also revealed that CRC patients had a significantly higher risk of dying from any of the non‐cancer‐related causes of death in comparison to the general US population (all *p* < 0.05). The only exceptions were syphilis and certain conditions originating in perinatal period (Figure 2C and Table S2).

### 3.3 Non‐cancer death of CRC patients by follow‐up time

**FIGURE 3:**
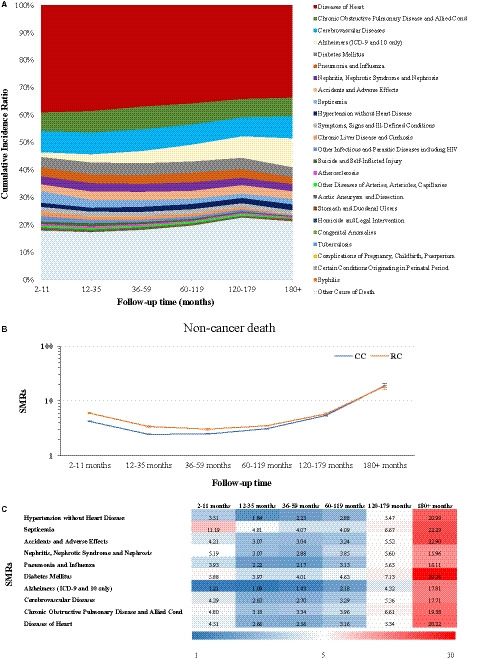
(A) The cumulative incidence ratio per cause of non‐cancer death cause of CRC patients by follow‐up time. (B) The SMR values of non‐cancer death cause for patients with CC and RC by follow‐up time. Horizontal lines are identified as SMR (middle) and 95% CI (upper: upper CI; lower: lower CI). (C) The SMR values of the 10 leading causes of non‐cancer death for CRC patients by follow‐up time.

According to follow‐up time, which corresponds directly to the survival time, the leading cause of non‐cancer death for CRC patients always remains heart disease; however, the cumulative incidence ratio gradually decreased from 38.97% for a follow‐up time of 2–11 months to 33.56% for the longest follow‐up time of ≥180 months (Figure 3A and Table S3). Similarly, decreasing relative distributions with increasing survival time were seen for septicemia (4.27% to 2.01%), chronic liver disease and cirrhosis (1.73% to 0.27%), other infectious and parasitic diseases including HIV (2.04% to 0.27%) as well as suicide and self‐inflicted injury (0.87% to 0.27%) (Figure 3A and Table S3).

On the contrary, with increasing survival time, CRC patients are more likely to die due to Alzheimer's disease (6‐fold increase from 1.73% with 2–11 months follow‐up time to 10.47% for at least 180 months follow‐up time) (Figure 3A and Table S3). Likewise, but less pronounced, hypertension without heart disease increased from 1.51% to 2.15% with increasing survival time as related non‐cancer death reason (Figure 3A and Table S3).

In general, the SMRs for non‐cancer death causes for both CC and RC patients were constantly higher than 1. Although the trend initially decreased, it increased with longer follow‐up time periods starting with the 12–35 months group for CC and with the 36–59 months group for RC (Figure 3B and Table S3).

The plotted SMRs for the 10 leading causes of non‐cancer death among CRC patients (detailed information for all causes of non‐cancer death are given in Table S3) can be obtained from Figure 3C. Albeit sometimes close to, still all SMR values were above 1 and displayed similar curve progressions. It is worth mentioning that among the 10 leading causes of non‐cancer‐related deaths, septicemia as reason for death has an over 11‐fold higher SMR (SMR, 11.19; 95% CI, 10.26–12.19; *p* < 0.05) within the first year after CRC diagnosis compared to the general US population. After at least 180 months follow‐up time, the highest and most significant SMR values were observed for diabetes mellitus (SMR, 29.28; 95% CI, 19.29–42.60; *p* < 0.05), followed by hypertension, accidents and adverse effects, septicemia as well as disease of heart with all SMRs >20 (all *p* < 0.05; Figure 3C and Table S3).

Moreover, we also calculated the SMRs of different follow‐up time periods for cause of non‐cancer death among subgroups of CRC patients (detailed information for all non‐cancer death causes among different subgroups of CRC patients in Table S4). Still similar with the entire cohort, but notably, CRC patients with distant metastasis showed the highest SMR values (SMR, 11.07; 95% CI, 10.73–11.42; *p* < 0.05), especially for 2–11 months follow‐up time (SMR, 24.43; 95% CI, 23.32–25.58; *p* < 0.05) (Table S4).

### 3.4 Non‐cancer death of CRC patients by age at diagnosis

**FIGURE 4:**
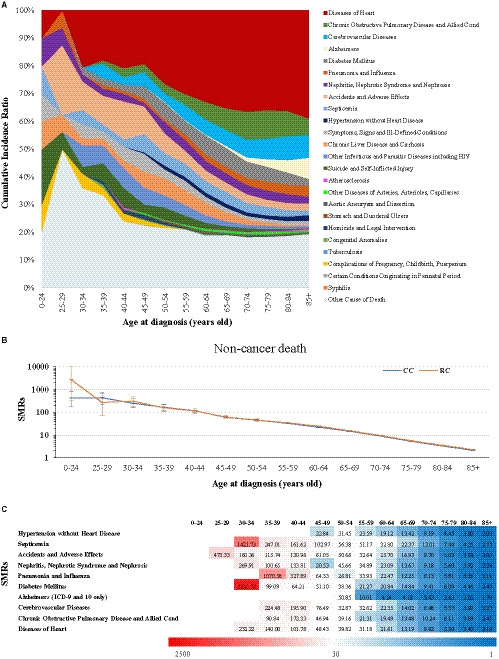
(A) The cumulative incidence ratio per cause of non‐cancer death for CRC patients with the increase of age at diagnosis. (B) The SMR values of non‐cancer death cause of patients with CC and RC with increase of age at diagnosis. Horizontal lines are identified as SMR (middle) and 95% CI (upper: upper CI; lower: lower CI). (C) The SMR values of the 10 leading causes of non‐cancer death for CRC patients with increase of age at diagnosis.

In total, and as could have been expected, we found that elder CRC patients had more common incidence of death from heart disease (over 30% within ≥55 years old group), chronic obstructive pulmonary disease and allied conditions (close to 10% among 70–84 years old group), cerebrovascular disease (over 8% among ≥80 years old), Alzheimer's disease (reach to 7.54% among ≥85 years old), pneumonia and influenza (near 4% among ≥85 years old) as well as hypertension without heart disease (2.10% for ≥85 year old) (Figure 4A and Table S5). In contrast, CRC patients younger than 50 years of age were more at risk to die from accidents and adverse effects (roughly 10%), other infectious and parasitic diseases including HIV (around 6%) as well as suicide and self‐inflicted injury (close to 5%; Figure 4A and Table S5).

Overall and in line with the preceding analyses, the SMRs for all non‐cancer deaths were for the different age groups always above 1 (Figure 4B). Moreover, a gradual decrease from young to old could be observed for CC and RC patients (Figure 4B). This result is frequently seen for the single non‐cancer death reasons (Figure 4C and Table S5). Of note, data could, for several death causes, not be given due to the lack of numbers of very young patients.

For patients younger than 50 years, SMR values over 100 were found for septicemia (Figure 4C and Table S5). Also, CRC patients with 30–34 years of age had an extremely high risk of death from diabetes mellitus as well as patients in the age group of 35–44, a very high risk for dying due to pneumonia and influenza (Figure 4C and Table S5).

When calculating SMRs for all non‐cancer death causes of the different subgroups and subdividing by age, very similar trends were continuously observed with SMRs being very high for the young patient groups to relatively low (but still higher than 2) for the older patients (Table S6).

## 4 DISCUSSION

The present study is the first report describing in detail the 25 causes of non‐cancer death of CRC patients in dependence from follow‐up time and patients’ age at diagnosis. A major finding was that during the period of 2000–2016, patients suffering from CRC were still at the highest risk to succumb to their disease, but this risk decreased from 70.19% to 49.35% during this relatively short period. Taking into account the large number of cases included into this analysis, one can now safely conclude that only about half of the newly diagnosed CRC patients are nowadays at risk to die directly due to their disease. On the downside, CRC patients’ death from non‐cancer causes has increased to 40.00% in 2016. While the SMRs for all non‐cancer death causes with the exception of syphilis and certain conditions originating in perinatal period are significantly above 1, the relative risk of non‐cancer death was for CRC patients always higher than the risk for the age‐matched general US population. This was especially pronounced for CRC patients with distant stage as well as the American Indians/Alaska Natives (AI/AN) ethnic subgroup (at least 10 times higher risk vs. general US population).

Zaorsky et al. reported a stable increase for all cancer patients’ death due to a non‐cancer cause since the 1970 s, and identified CRC as one of those cancer entities least likely directly causing patients’ death.^9^ Over the last decades, the survival rates of patients with CRC have significantly improved in the developed world. The resulting decreased mortality can be attributed to better treatment modalities.^18^ The results of the present study demonstrate that this is fortunately a still ongoing development and, consequently, more and more patients die from non‐CRC causes.

The single leading non‐cancer death cause accounting for approximately one‐third of CRC patients’ death is a condition of heart disease, followed by chronic obstructive pulmonary disease as well as cerebrovascular diseases with almost identical frequencies (7.57% and 7.55%, respectively). These findings are not surprising when taking into consideration that heart disease is generally the first leading cause of death in the US, followed by cancer, chronic lower respiratory diseases, and cerebrovascular diseases (i.e., stroke) as the fifth leading cause of death.^19^ In addition, this proportion of death from cardiac, chronic obstructive pulmonary, and cerebrovascular diseases can, for the most part, be attributed to the fact that most CRC patients are elder. Besides the abovementioned death reasons, pneumonia and influenza, hypertension as well as atherosclerosis have in the present study been found to be significantly more frequent in older CRC patients in comparison to not only the overall US population but also to the younger aged CRC patient groups. Dong Liu et al. reported on the continuous increase of, especially, cardiovascular disease but also hypertension, heart failure, and atrial fibrillation in cancer patients with old age.^20^ This adds to the fact that aging itself is a risk factor for cancer development.^21^, ^22^ However, this analysis revealed that CRC patients’ relative risk of dying due to cardiac and cerebrovascular diseases is also constantly higher when compared to the age‐matched general population. One might speculate that this is likely attributable to a constricted fitness of most cancer patients ^23^, ^24^ as well as shared risk factors between CRC development and, for example, cardiovascular disease.^25^, ^26^ Therefore, increasing numbers of CRC patients have concomitant risk factors for cardiac, chronic lower respiratory, and cerebrovascular diseases at time of diagnosis. A Spain population‐based cohort study reported that when patients suffer from congestive heart failure combined with diabetes, the most frequent co‐diseases of CRC patients, this sums up for a higher short‐term mortality risk.^27^

Another malignant condition clearly associated with aging is Alzheimer's disease.^19^ We observed that up to 10.47% of the long‐term surviving CRC patients died due to Alzheimer's disease with stably increasing SMRs with follow‐up time. The fact that SMRs of Alzheimer's disease were rising with the extension of follow‐up time in all cancer patients has been reported before.^9^ In contrast, but within the expectations, a relatively high proportion of patients died within the first year after CRC diagnosis of septicemia and infectious diseases, attributable to postoperative infectious complications.^28^, ^29^ As discussed before, the comorbidities, which most elderly CRC patients have, may increase their risk of peri‐ and postoperative mortality.^30^, ^31^ Nowadays, surgery is still the most common therapy for CRC; however, RC surgery even has higher morbidity or/and mortality as well as local relapse rates in comparison to CC operation.^32^ In line with this, the present study also observed higher SMRs for RC compared to CC patients.

Contrary to the discussed death reasons associated with older age observed in the groups of 50 years or older of the CRC patients, the present study could confirm previous results that accidents and adverse effects, non‐septic infectious diseases, and suicide are more common reasons of death for CRC patients younger than 50 years.^9^ Of note, the SMRs of the top 10 leading non‐cancer death causes were higher for younger than for older CRC patients. In other words, the relative risk of non‐cancer death in comparison to the corresponding general US population is highest for young CRC patients. And this augmented risk shrinks with increasing age. This might be partly explained by the fact that, despite the decrease of CRC incidence and mortality in general, for the past decade, an increase of both in patients younger than 50 years has been observed.^1^, ^33^ When additionally taking into account that cancer patients younger than 50 years have a 1.8‐fold higher risk of psychological distress compared to patients >50 years of age,^34^ we would like to conclude that, especially, in the clinical management of younger CRC patients, psychological guidance should be emphasized.

In addition, we observed that the relative death risk from non‐cancer causes was higher for patients with only 2–11 months follow‐up time. It is a well‐established fact that chemotherapy is associated with better cancer survivorship on the one hand, but on the other hand, patients suffer from the chemotherapy‐associated toxicities with some even showing long‐term side effects including heart and lung diseases.^35^, ^36^, ^37^, ^38^ The situation is similar for radiotherapy in RC.^39^ Another unwanted effect of the antineoplastic treatments is possibly an increase in blood pressure values.^38^ Also targeted and immune‐therapeutics, which play a rapidly increasing role in CRC management, have been associated with long‐term side effects including pneumonitis,^40^, ^41^ hypertension,^41^, ^42^ and blood clotting.^43^ This multitude of serious therapy‐associated side effects are thus likely causative for non‐cancer deaths occurring close to the time point of multi‐therapeutic management.

Patients with distant CRC have a very poor survival outcome,^44^ making a serious disease course a likely reason for patients’ death from CRC itself. Interestingly, the subgroup analysis revealed a significantly higher death risk from non‐cancer causes of CRC patients with distant stage versus the general US population, particularly in the first year of follow‐up time. Most prominent were septicemia as well as other infections and parasitic diseases, which can, as mentioned above, likely be attributed to postoperative infectious complications, supporting previous results.^29^ In addition, CRC patients with AI/AN ethnical background have a significantly higher risk of non‐cancer death with death due to hypertension being most likely for this subgroup when compared with the general US population. In sum, these findings suggest that an improvement in the clinical and early post‐clinical management of infections and hypertension has a high potential to prevent CRC patient deaths.

One limitation of the present study comes from the fact that for 19.07% of patients, their death could not be attributed to a single cause and have thus crudely been named as other causes of death. This makes a bias such as an underestimation of some death causes likely. Moreover, and as discussed, some non‐cancer death causes can closely be associated with therapeutic measures such as chemotherapy but the SEER database does not contain complete data of CRC patients’ therapeutical management. Lastly, some SMR values for several death causes are not computable due to the lack in numbers of very young patients.

## 5 CONCLUSION

In conclusion, CRC patient's risk of succumbing to their malignant disease is rapidly decreasing, but is of course accompanied by a counter wise gradual increase in non‐cancer causes of death. Heart disease was the most common cause of non‐cancer death after CRC diagnosis during the period of 2000–2016. Different proportions of non‐cancer deaths were observed in dependence from patient's survival time and age at diagnosis. Most pronounced was a high proportion of CRC patients dying of cardiac, chronic lower respiratory, and cerebrovascular diseases, an increasing proportion of CRC patients succumbing to Alzheimer's disease with an increase in survival time, and a very high proportion of younger CRC patients dying from accidents and adverse effects, non‐septic infections as well as suicide. Also, the higher risk of non‐cancer death should be noted when compared with general US population within the first year after CRC diagnosis. This observation is especially true for the younger patient groups as well as CRC subgroups of distant stage and AI/AN ethnicity. Since at least some of the non‐cancer death reasons could eventually result from long‐term therapeutical side effects, the findings of the present work could help to modify and sharpen strategies to improve clinical management and treatment of CRC patients. It moreover emphasizes the great need for clinical research to enhance cancer patients’ quality of life and extend their meaningful survival time.

## CONFLICT OF INTEREST

The authors have no conflict of interest to disclose.

## FUNDING INFORMATION

L.L was supported by the China Scholarship Council (grant number: 201908080127).

## Supporting information

Table S1

Table S2

Table S3

Table S4

Table S5

Table S6

## Data Availability

The data from SEER (https://seer.cancer.gov) are available in present study.
